# Association of HLA-B27 status and gender with sacroiliitis in patients with ankylosing spondylitis

**DOI:** 10.12669/pjms.301.3896

**Published:** 2014

**Authors:** Jiangbiao Xiong, Jing Chen, Jianxin Tu, Wenjing Ye, Zhiyong Zhang, Qiaoqiong Liu, Xiaochun Zhu

**Affiliations:** 1 Jiangbiao Xiong, Department of Rheumatology, the First Affiliated Hospital of Wenzhou Medical University, Wenzhou 325035, PR China.; 2 Jing Chen, Department of Rheumatology, the First Affiliated Hospital of Wenzhou Medical University, Wenzhou 325035, PR China.; 3 Jianxin Tu, Department of Rheumatology, the First Affiliated Hospital of Wenzhou Medical University, Wenzhou 325035, PR China.; 4 Wenjing Ye, Department of Rheumatology, the First Affiliated Hospital of Wenzhou Medical University, Wenzhou 325035, PR China.; 5 Zhiyong Zhang, Department of Rheumatology, the First Affiliated Hospital of Wenzhou Medical University, Wenzhou 325035, PR China.; 6 Qiaoqiong Liu, Department of Rheumatology, the First Affiliated Hospital of Wenzhou Medical University, Wenzhou 325035, PR China.; 7 Xiaochun Zhu, Department of Rheumatology, the First Affiliated Hospital of Wenzhou Medical University, Wenzhou 325035, PR China.

**Keywords:** Ankylosing spondylitis, HLA-B27, Gender, Sacroiliitis, Computed tomography

## Abstract

***Objective: ***To observe the influence of human leucocyte antigen B27 (HLA-B27) status and gender on sacroiliitis on computed tomography (CT) in ankylosingspondylitis (AS).

***Methods: ***We reviewed the archived medical records of the AS inpatients admitted in the Rheumatology Department of the First Affiliated Hospital of Wenzhou Medical University during the period from January 2007 through January 2013 and finally 386 patients were included in the study. The severity of sacroiliitis on CT was evaluated according to the grading used in the modified New York criteria for AS. Two-way classification analysis of variance (ANOVA) was employed to examine the effect of HLA-B27 status and gender on age at disease onset. The impact of HLA-B27 and gender on sacroiliitis on CT was tested by univariate and multivariate logistic regression analyses.

***Results: ***There were 350 HLA-B27 positive patients (90.7%) and 36 HLA-B27 negative patients (9.3%). The ANOVA test indicated that HLA-B27 positive patients and male patients respectively had an earlier age at disease onset than HLA-B27 negative patients and female patients. The logistic regression analysis indicated that positive HLA-B27 status (OR 2.601, p=0.004) and male gender (OR 1.923, p=0.004) were significant predictors of worse sacroiliitis. In addition, elevated ESR (OR 2.181, p=0.013) and longer disease duration (OR 1.100, p<0.001) contributed to worse sacroiliitis likewise.

***Conclusion: ***Positive HLA-B27 status and male gender are associated with worse sacroiliitis on CT, acting as predictors of sacroiliitis. Elevated ESR and longer disease duration also contribute to worse sacroiliitis. Meanwhile, positive HLA-B27 status and male gender are associated with earlier age at disease onset.

## INTRODUCTION

Ankylosingspondylitis (AS) is a progressive chronic inflammatory disease that affects the axial skeleton, leading to structural damage and functional limitation.^1^ Clinical manifestations of AS usually begin in late adolescence or early adulthood; only rarely do they begin after age 40 years. The characteristic clinical symptoms of AS are inflammatory back pain (IBP) and morning stiffness, whereas they are often not well recognized at first.^2^ Usually, sacroiliac (SI) joint is the first joint involved, and computed tomography (CT) has been used for evaluation of sacroiliitis since 1979 for its higher diagnostic accuracy than radiography.^3^^-^^5^ Apart from IBP and sacroiliitis, various clinical manifestations, including spondylitis, hip or peripheral joint arthritis, enthesitis, and uveitis, can occur in the course of disease.^1^

Another important diagnostic clue for AS is human leukocyte antigen B27(HLA-B27), whose association with AS is one of the strongest associations between HLA molecules and disease.^6^^,^^7^ Literature reported that about 90% of white patients with AS possessed positive HLA-B27 status. A recent nationwide epidemiological investigation in China showed that approximately 78.4% of AS patients were diagnosed asHLA-B27 positive carriers，however, the exact role of HLA-B27 in AS is still unknown.^6^^, ^^8^^-^^10^ Apart from the contribution of HLA-B27 factor, there have been some studies suggesting that non-HLA genetic factors and bowel inflammation play a role in pathogenesis of AS.^11^^-^^14^ Furthermore, it has been reported that HLA-B27 positive and male patients are different from HLA-B27 negative and female patients in some characters, such as joints involvement, age at disease onset, disease activity and anterior uveitis.^15^^-^^19^

This study aimed to estimate the influence of HLA-B27 status and gender on sacroiliitis on CT. To our knowledge there were few reports investigating about the relationship between HLA-B27 status and severity of sacroiliitis on CT imaging in AS, and our study focused on that.

## METHODS

***Patients: ***All the archived medical records of the inpatients admitted in the Rheumatology Department of the First Affiliated Hospital of Wenzhou Medical University during the period from January 2007 through January 2013 were retrospectively investigated. Four hundred and thirty-one patients definitely diagnosed as AS according to the 1984 modified New York criteria for AS were chosen to participate in this study.^20^ Twenty one patients who had incomplete records about laboratory examination and 24 patients who had no records about CT images of SI joints were excluded from this study. Written informed consent was obtained from the patient individually for publication of this report. The study was approved by the Ethics Committee of the First Affiliated Hospital of Wenzhou Medical University, Wenzhou, China.

All the archived medical records of the patients were reviewed, and the diagnoses were reconsidered by two experienced rheumatologist independently. The diagnoses, age, sex, disease duration, age at disease onset, HLA-B27 status, *erythrocyte sedimentation rate (ESR), C-reactive protein (CRP), serum *immunoglobulin A (IgA) and platelet count of all the AS patients were recorded at the same time. Only patients with consistent diagnoses from the two rheumatologists were enrolled into the final statistical process.

**Fig.1:**
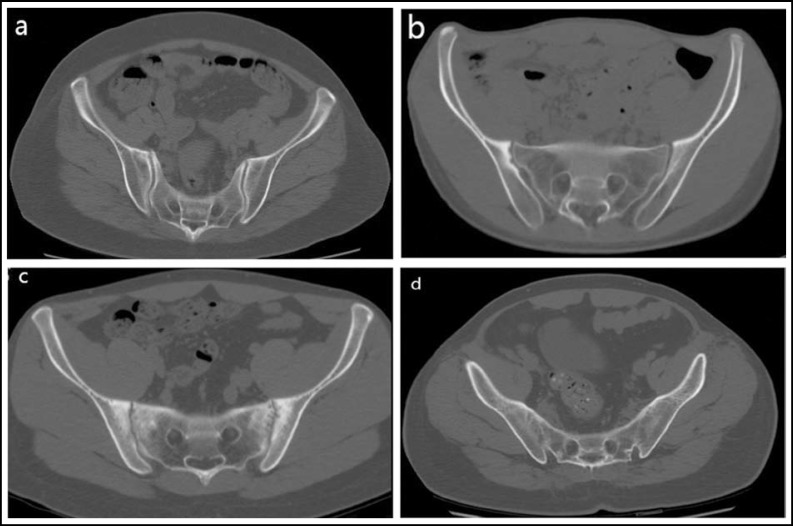
Grading of sacroiliitis is shown on the axial image of CT by using the bone window setting. (a) grade 1 shows suspicious changes, (b) grade 2 shows small localized areas with erosion or sclerosis, (c) grade 3 shows definite abnormality with erosions, sclerosis, joint space widening or narrowing, (d) grade 4 shows complete ankylosis

***CT imaging: ***All the patients had taken pelvic CT scanning examinations that were processed using the bone window setting (Fig.1). The CT images were evaluated independently by two trained radiologists who were blinded to the patients’ clinical complaints and diagnoses. The severity of sacroiliitis was scored according to the grading used in the modified New York criteria for AS as follows: Grade 0 = normal; Grade 1 = suspicious changes; Grade 2 = minimal abnormality (small localized areas with erosion or sclerosis, without alteration in the joint width); Grade 3 = unequivocal abnormality (moderate or advanced sacroiliitis with one or more of erosions, evidence of sclerosis, widening, narrowing, or partial ankylosis); Grade 4 = severe abnormality (total ankylosis). If the grades of left and right SI joint in one patient were unequal, the higher grade one was chosen as evaluating severity of sacroiliitis. When two radiological assessors did not agree with the radiological evaluation of sacroiliitis, the end result was defined by a consensus between the investigators.

***Statistical Analysis: ***SPSS Version 18.0 was used for data analysis. The significance of percentage differences was determined by the chi-square test for 2x2 table, or Fisher probabilities in 2x2 table if necessary. Where average values or standard deviations of age indications were compared, the significance was tested by the *t* test or, where appropriate, the F test. For the effect of HLA-B27 status and gender on age at disease onset, we employed the two-way classification analysis of variance (ANOVA) test. Factors in relation to sacroiliitis on CT were assessed using different univariate and multivariate logistic regression analyses. Significant independent variables in univariate analyses were retested in multivariate regression. All statistical tests were two-sided, and P <0.05 was considered statistically significant.

## RESULTS

**Fig.2:**
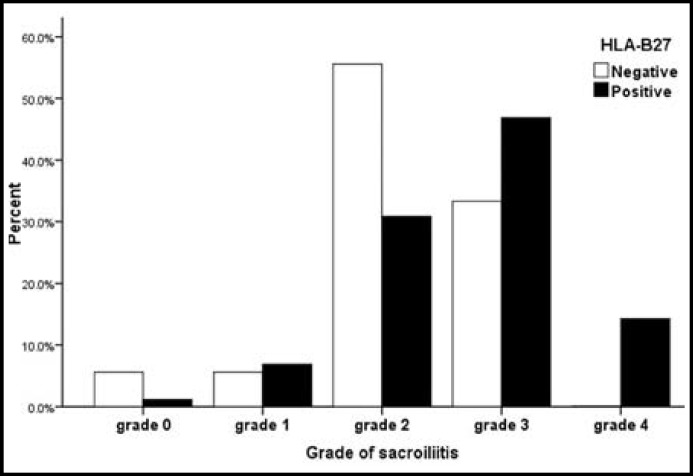
Percentage of HLA-B27 status in relation to grade of sacroiliitis

After screening the archived medical records according to the diagnostic criteria and relevant exclusion standards mentioned above, a total of 386 patients (284 men and 102 women, median age 35 years, range 14-73 years, Table-I) with AS were finally enrolled in this study. Among the 386 AS patients, 350 patients (90.7%) were HLA-B27 positive and 36 patients (9.3%) were HLA-B27 negative. Laboratory variables reflecting the inflammation were also listed in Table-I. The median disease duration was 4 years (range 0.1–40.3 years), and no statistical difference was seen in different gender(male 5.6±6.0 years, female 5.5±7.5 years; p=0.819) and HLA-B27 status (positivity 5.7±6.6 years, negativity 4.7±3.8 years; p=0.372). The median age at disease onset (occurrence of first AS symptoms) of the study group was 27 years (range 5–72 years). The sacroiliitis of 386 patients were evaluated according to the grading used in the modified New York criteria for AS mentioned above (Fig.1). Grade 0 was assigned in 5 patients, grades 1 in 26 patients, grade 2 in 129 patients, grades 3 in 176 patients, and grade 4 in 50 patients (Table-I). As showed in Fig. 2, HLA-B27 negative patients (55.6%) mainly lay in grade 2, and HLA-B27 positive patients (46.9%) mainly lay in grade 3.

The ANOVA test (Table-II) indicated that HLA-B27 positive patients (29.9±13.1 years)and male patients (28.5±13.0 years) respectively had an earlier age at disease onset than HLA-B27 negative patients (34.5±12.1 years)and female patients (35.4±11.9 years). There was a higher prevalence of juvenile disease onset (age at disease onset<16 years) in male patients than in female patients (12.3% vs. 2.9%, respectively; p=0.006), and no significant difference was seen between HLA-B27 positive patients and HLA-B27 negative patients (10.3% vs. 5.6%, respectively; p=0.540).On the other hand, the disease onset after 40 years of age was significantly more common in females than in males (35.3% vs. 15.5%, respectively; p<0.001), and no significant difference was seen between HLA-B27 positive patients and HLA-B27 negative patients (20.0% vs. 27.8%, respectively; p=0.273).

The correlations between the severity of sacroiliitis on CT and clinical parameters are listed in Table-III. HLA-B27 positivity, male gender, elevated ESR, elevated CRP and disease duration in univariate analyses showed a p value of <0.05, and were therefore retested in multivariate logistic regression analysis. The multivariate logistic regression analysis indicated that positive HLA-B27 status (OR 2.601, p=0.004) and male gender (OR 1.923, p=0.004) were significant predictors of worse sacroiliitis. In addition, elevated ESR (OR 2.181, p=0.013) and longer disease duration (OR 1.100, p<0.001) contributed to worse sacroiliitis likewise.

## DISCUSSION

From the results above, we could find that positive HLA-B27 status and male gender were associated with earlier age at disease onset. Importantly, positive HLA-B27 status and male gender were associated with worse sacroiliitis on CT. Elevated ESR and longer disease duration also contributed to worse sacroiliitis.

**Table-I:** Characteristics of 386 AS patients included in the study

*Median age (years)*	*35 (14-73)*
Male gender	284 (73.6%)
HLA-B27 positivity	350 (90.7%)
Median disease duration(years)	4 (0.1-40.3)
Median age at disease onset(years)	27 (5-72)
No. of patients in each grade of sacroiliitis	
Grade 0	5
Grade 1	26
Grade 2	129
Grade 3	176
Grade 4	50
Median ESR (mm/h)	46 (2-120)
Percentage of patients with elevated ESR	76.7
Median CRP (mg/L)	18.3 (1.0-178.0)
Percentage of patients with elevated CRP	72.5
Median serum IgA (g/L)	3.01 (0.67-8.43)
Percentage of patients with elevated IgA	17.1
Median platelet (x10^9^/L)	274 (99-584)
Percentage of patients with elevated platelet	37.8

*HLA-B27, *human leucocyte antigen B27; *ESR, erythrocyte sedimentation rate;*

*CRP,* *C-reactive protein; IgA, *immunoglobulin A.

**Table-II:** Two-way classification ANOVA for examining the effect of HLA-B27 status and gender on age at disease onset

	*Age at disease onset* *(years)*	*P value*
HLA-B27positivity	29.9±13.1	0.042
HLA-B27 negativity	34.5±12.1	0.042
Male gender	28.5±13.0	<0.001
Female gender	35.4±11.9	<0.001

ANOVA, analysis of variance; *HLA-B27, *human leucocyte antigen B27

**Table-III:** Correlations between clinical parameters and the grade of sacroiliitis on CT

*Logistic regression*	*OR (95% CI)*	*P value*
Univariate analysis		
HLA-B27 positivity	2.821 (1.480-5.376)	0.002
Male gender	1.655 (1.084-2.527)	0.019
Elevated ESR	1.896 (1.219-2.954)	0.005
Elevated CRP	1.584 (1.043-2.404)	0.031
Elevated IgA	1.163 (0.709-1.906)	0.551
Elevated platelet	1.372 (0.933-2.016)	0.107
Disease duration	1.097 (1.063-1.133)	<0.001
Multivariate analysis		
HLA-B27 positivity	2.601 (1.357-4.988)	0.004
Male gender	1.923 (1.228-3.016)	0.004
Elevated ESR	2.181 (1.179-4.035)	0.013
Elevated CRP	1.055 (0.598-1.866)	0.852
Disease duration	1.100 (1.065-1.135)	<0.001

*OR, odds ratio; 95% CI, 95% confidence interval* *; HLA-B27, *human leucocyte antigen B27;

*ESR,* *erythrocyte sedimentation rate* *; CRP,* *C-reactive protein; IgA, *immunoglobulin A.

As is known, AS patients are often HLA-B27 positive carriers. In our study, the prevalence of positive HLA-B27 status was 90.7%. Also, AS is more common in men, with a reported male-to-female ratio of about 2:1 to 3:1, probably as a result of under diagnosis in women. In fact, female AS patients may represent a largely under diagnosed and understudied population. The reasons for this under diagnosis could include a continuing bias of AS being a disease of only men or differences in the disease expression in women that could cause delayed or missed diagnoses.^21^ Generally, disease expression is thought to be different according to HLA-B27 status and gender. Previous studies were focused on differences in joints involvement, age at disease onset, disease activity and anterior uveitis. However, the exact role of gender and HLA-B27 in pathogenesis of AS is still unknown.

The impact of HLA-B27status and gender on age at disease onset has been reported in some studies.^16^^, ^^17^^,^^19^^, ^^22^^-^^24^ Our study showed HLA-B27 positive patients and male patients respectively had a significantly earlier age at disease onset than HLA-B27 negative patients and female patients, which was consistent with previous studies. We found a significantly higher prevalence of juvenile disease onset (age at disease onset<16 years) in male patients than in female patients, and no significant difference in different HLA-B27 status, consistent with a recent study showing no significant difference in terms of HLA-B27 status.^10^ Although clinical symptoms of AS usually begin in late adolescence or early adulthood, late onset AS (after 40 years of age) does occur. Moreover, the frequency of late disease onset is significantly higher in female patients, but no statistical difference was found in different HLA-B27 status, not consistent with Feldtkeller’s results.^17^ In addition, it has been reported that the average delay between the first spondyloarthritic symptoms and the diagnosis in HLA-B27 positive patients and male patients is significantly longer than inHLA-B27 negative patients and female patients.^13^^,^^14^^,^^21^^, ^^17^^, ^^16^ 

AS is a chronic inflammatory disease, generally beginning with inflammation in SI joints. Elevated ESR, CRP, serum IgA and platelet count standing for inflammation are often seen in AS patients. Proof of sacroiliitis on imaging is important for diagnosis in modified New York criteria for AS. Though plain pelvic radiograph is still conventional for the evaluation of SI joints in patients suspected of AS, its role in diagnosis and evaluation of sacroiliitis is being challenged by CT. Plain pelvic radiograph lacks sensitivity in early sacroiliitis, whereas CT scan can detect bone abnormalities such as sclerosis and erosion sooner than plain radiography.^3^^,^^5^^,^^25^ According to modified New York criteria for AS, HLA-B27 positivity is not necessary for diagnosis. However, HLA-B27 status does have an impact on sacroiliitis was the conclusion from this study. Our logistic regression analysisindicatedHLA-B27 positivity and male gender were independently associated with worse sacroiliitis on CT imaging, suggesting that HLA-B27 status and gender played as predictors of worse sacroiliitis in AS. In our study, the majority of HLA-B27 negative patients (55.6%) lay in grade 2sacroiliitis, whereas HLA-B27 positive patients (46.9%) mainly lay in grade 3. Previous studies concentrated on radiographic sacroiliitis, and they found no difference in terms of HLA-B27 status and gender.^26^^, ^^27^ That might result from poor sensitivity of radiography for evaluating sacroiliitis and their small sample size. The recent study of Ho Yin Chung et al showed that HLA-B27 positivity and male gender were associated with inflammation of SI joints on magnetic resonance imaging (MRI) and that HLA-B27 was associated with skeletal damage of the SI joints.^16^ They thought HLA-B27 status and gender might have prognostic importance to structural damage in spondyloarthritis. Finally, we noticed that elevated ESR and longer disease duration contributed to worse sacroiliitis, which was in accordance with the common view that AS is a chronic inflammatory disease progressing over time.

Our study has several limitations. First, we took the New York radiological grading criteria to evaluate sacroiliitis on CT images, which is not entirely appropriate and ideal.^28^ Second, we didn’t take HLA-B27 subtypes into account. There have been reports showing that several HLA-B27 subtypes (e.g. B2706 and B2707)might be protective factors of AS, especially in Asia.^29^^-^^31^ So, further study need to specially evaluate the effect of HLA-B27 subtypes on sacroiliitis. Third, the sample size of HLA-B27 negative patients in our study is still not large enough.

In conclusion, positive HLA-B27 status and male gender are associated with worse sacroiliitis on CT, acting as predictors of worse sacroiliitis. Elevated ESR and longer disease duration also contribute to worse sacroiliitis. Meanwhile, positive HLA-B27 status and male gender are associated with earlier age at disease onset.

## Author contributions:

**Jiangbiao Xiong:** General design, statistical analysis, writing the manuscript.

**Jing Chen, Jianxin Tu, Wenjing Ye, Zhiyong Zhang and Qiaoqiong Liu:** Data acquisition and data analysis. 

**Xiaochun Zhu:** General design, guaranteeing integrity of the study, manuscript editing.
